# Sulfonylureas for Treatment of Periodontitis-Diabetes Comorbidity-Related Complications: Killing Two Birds With One Stone

**DOI:** 10.3389/fphar.2021.728458

**Published:** 2021-09-01

**Authors:** Luxi Yang, Qing Ge, Zhitong Ye, Lijing Wang, Liping Wang, Mubarak Ahmed Mashrah, Janak L. Pathak

**Affiliations:** ^1^ Guangzhou Key Laboratory of Basic and Applied Research of Oral Regenerative Medicine, Affiliated Stomatology Hospital of Guangzhou Medical University, Guangzhou, China; ^2^ School of Life Sciences and Biopharmaceutics, Vascular Biology Research Institute, Guangdong Pharmaceutical University, Guangzhou, China

**Keywords:** periodontitis-diabetes comorbidity, sulfonylureas, inflammation, bone metabolism, angiogenesis

## Abstract

Periodontitis is one of the most prevalent oral inflammatory diseases leading to teeth loss and oral health problems in adults. Periodontitis mainly affects periodontal tissue by affecting the host immune system and bone homeostasis. Moreover, periodontitis is associated with various systemic diseases. Diabetes is a metabolic disease with systemic effects. Both periodontitis and diabetes are common inflammatory diseases, and comorbidity of two diseases is linked to exacerbation of the pathophysiology of both diseases. Since bacterial dysbiosis is mainly responsible for periodontitis, antibiotics are widely used drugs to treat periodontitis in clinics. However, the outcomes of antibiotic treatments in periodontitis are not satisfactory. Therefore, the application of anti-inflammatory drugs in combination with antibiotics could be a treatment option for periodontitis-diabetes comorbidity. Anti-diabetic drugs usually have anti-inflammatory properties and have shown beneficial effects on periodontitis. Sulfonylureas, insulin secretagogues, are the earliest and most widely used oral hypoglycemic drugs used for type-2 diabetes. Studies have found that sulfonylurea drugs can play a certain role in the mitigation of periodontitis and inflammation. This article reviews the effects of sulfonylurea drugs on the mitigation of periodontitis-diabetes comorbidity-related inflammation, bone loss, and vascular growth as well as the involved molecular mechanisms. We discuss the possibility of a new application of sulfonylureas (old drug) to treat periodontitis-diabetes comorbidity.

## Introduction

Periodontitis refers to the chronic inflammation of tooth-supporting tissues. The loss of periodontal attachment, surrounding bone, and teeth are clinical features of periodontitis (Gasner and Schure, 2020). According to the fourth national oral health epidemiological survey, almost 90% of Chinese adults suffered from periodontal disease of various severities and >30% of them had severe periodontitis (Jiao et al., 2021). Periodontitis has a high incidence rate and the prevalence of severe periodontitis in the world has reached approximately 10% (Frencken et al., 2017). In 2019, there were 1.1 billion (95% uncertainty interval: 0.8–1.4 billion) prevalent cases of severe periodontitis globally. From 1990 to 2019, the age-standardized prevalence rate of severe periodontitis increased by 8.44% (6.62–10.59%) worldwide (Chen et al., 2021). Shreds of evidence indicate that periodontitis is associated with many systemic diseases, including cardiovascular disease, type 2 diabetes mellitus (T2DM), rheumatoid arthritis, inflammatory bowel disease (IBD), Alzheimer’s disease, nonalcoholic fatty liver disease, and certain cancers (Hajishengallis and Chavakis, 2021). Periodontitis leads to a 19% increase in the risk of cardiovascular disease (Nazir, 2017). Oral inflammatory diseases aggravate chronic inflammatory lung diseases such as asthma and chronic obstructive pulmonary disease (COPD) (Coffee and Sockrider, 2019). There are many common risk factors between periodontitis and osteoporosis, and the two influence each other (Wang and McCauley, 2016). Periodontitis is also associated with maternal infections, premature birth, low birth weight, and pre-eclampsia (Nazir, 2017). Therefore, periodontitis is not only an oral health problem but has a direct systemic connection and aggravates systemic inflammatory diseases. Due to the unique comorbidities and exacerbation relation between periodontitis and various systemic inflammatory diseases, periodontitis has become a huge public health burden.

**FIGURE 1:**
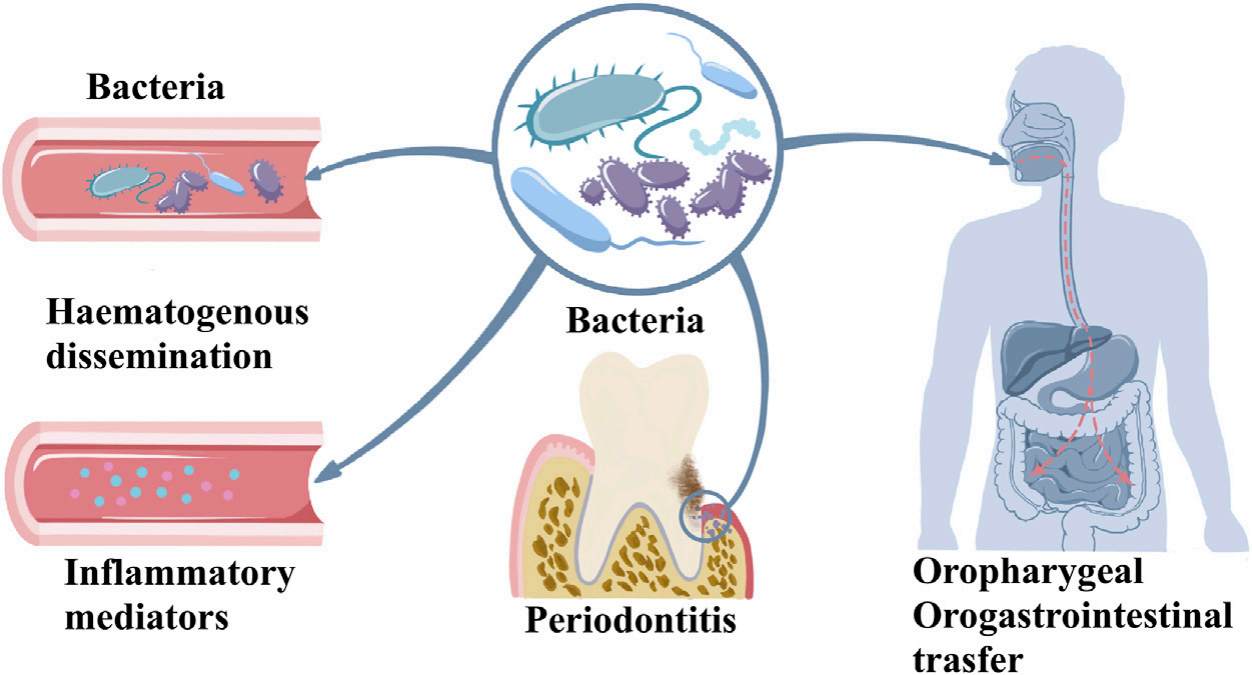
The possible route of systemic effects of periodontitis.

Plaque microorganisms play an indispensable role in the pathogenesis of periodontitis, but their existence alone does not mean that periodontitis will inevitably occur. The combined action of some risk factors eventually leads to the occurrence of periodontitis (Darveau, 2010; Hajishengallis, 2014). In periodontitis, oral microbiome dysbiosis triggers host response inducing inflammatory mediators in oral tissues. This effect causes the periodontitis and oral tissue degeneration. Oral bacteria and inflammatory mediators enter the circulation exerting systemic effects. Moreover, oral microbiome can directly enter to oropharyngeal and orogastrointestinal route that further causes systemic effect as indicated in Figure 1. Over the past 30 years, a large number of research results have proved the host’s defense response, or the host’s susceptibility to periodontal disease as a necessary condition to develop disease (Darveau, 2010). The multi-microbial synergy and biological disorders of susceptible hosts are the initiation events of periodontitis. The host’s defense barrier and defense cells have anti-inflammatory or pro-inflammatory. As the host’s first line of defense against pathogenic microorganisms, neutrophils not only engulf bacteria through their Fc receptor-binding antibodies but also release collagenase, which has a destructive effect on tissues (Hajishengallis, 2020). *Porphyromonas gingivalis*, the main pathogen of periodontitis, polarizes macrophages to the M1 phenotype and their penetration into the gum tissue also leads to alveolar bone resorption (Zhu et al., 2019). Compared with healthy tissues, the proportion of monocytes observed in periodontitis tissue is significantly higher, and these monocytes overexpress human leukocyte antigen-DR (HLA-DR) and programmed cell death 1 ligand-1 (PDL-1) molecules, indicating that monocytes are in an inflammatory state during periodontitis. The ratio of M1/M2 macrophages is also significantly higher at the site of periodontal infection, indicating an imbalance between inflammation and repair mechanisms (Almubarak et al., 2020). Actinobacillus actinomycetemcomitans, a Gram-negative anaerobic microorganism that causes periodontitis in humans, activates CD4^+^ cells in periodontitis tissues and cause local alveolar bone destruction (Teng et al., 2000). Immune cells release a large number of cytokines, such as interleukin-1β (IL-1β), IL-6, IL-8, tumor necrosis factor-α (TNF-α), prostaglandin-E2 (PGE2), matrix metalloproteinases (MMPs), interferon-γ (IFN-γ), and receptor activator of nuclear factor-κB ligand (RANKL) in response to bacterial invasion. These cytokines respective effects causing tissue damage to the periodontal region (Ramadan et al., 2020). Under physiological conditions, bone remodeling is the result of coordination between bone formation and resorption. The balance between osteoblast-mediated bone formation and osteoclast-mediated bone resorption ensures bone homeostasis. In an inflammatory state, excessive activation of the immune system and increased pro-inflammatory factors promote RANKL-mediated osteoclast formation, leading to bone resorption (Jung et al., 2014; Renn et al., 2018). At the same time, the host’s susceptibility factors, including genetic factors, endocrine, immune function, psychological regulation, and certain diseases such as diabetes, are important in the development and aggravation of periodontitis (Badran et al., 2015). Both diabetes and periodontitis are systemic inflammatory diseases that accelerate the development and progression of each other. Comorbidity of the two diseases is associated with poor health and quality of life.

## Diabetes

Diabetes is a chronic metabolic disease. In the past few decades, the prevalence of diabetes has increased significantly in the world. More than 422 million adults worldwide had diabetes in 2014 (Collaboration, 2016). Diabetes is categorized as type 1 diabetes mellitus (T1DM), T2DM, and pregnancy diabetes. T1DM is an autoimmune disease characterized by the destruction of pancreatic β-cells. T2DM is characterized by insulin resistance and impaired insulin secretion. The ability of T2DM patients to produce insulin in the body is not completely lost, and this type of diabetes treated with certain oral drugs that stimulate insulin secretion in the body. Gestational diabetes refers to a situation where women without a history of diabetes have elevated blood sugar during pregnancy. In this case, the baby has a high risk of developing diabetes in adulthood (Lovic et al., 2020). The global burden of diabetes is increasing with the increasing incidence rate of the disease.

## Diabetes and Periodontitis Comorbidity

The prevalence of diabetes-affected people worldwide is expected to be 642 million by 2040 (Ogurtsova et al., 2017). Periodontitis is closely related to the progression of diabetes. There was sufficient evidence in the mid-1990s that regards periodontitis as the sixth complication of diabetes (Loe, 1993). New consensus indicates periodontal disease and diabetes as comorbidity (American Diabetes, 2018). Periodontitis is closely related to the aggravation of diabetes and T2DM is regarded as the risk factor of periodontitis (Chávarry et al., 2009). Similarly, the severity of periodontitis affects the blood sugar control of diabetic patients and exacerbates diabetes-related complications (Lalla and Papapanou, 2011). Multiple meta-analysis studies have shown that periodontal treatment alone can have a beneficial effect on glycemic control (Simpson et al., 2010; Teeuw et al., 2010; Sgolastra et al., 2013). On the other hand, anti-diabetic drugs not only play an indirect role in treating inflammation due to diabetes control but also have a direct anti-inflammatory effect (Kothari et al., 2016). The anti-inflammatory properties of anti-diabetic drugs have the potential to ameliorate periodontitis. At the same time, prospective studies had shown that patients with moderate or severe periodontitis have a significantly increased risk of diabetes in the future (Morita et al., 2012). Therefore, the exploration of a drug that can treat periodontitis-diabetes comorbidity could be of clinical significance.

As aforementioned, the clinical observation that periodontitis and diabetes can interact with each other is well researched. The mechanism of the association between these two diseases is still under study, but there are some accepted theories. It is generally believed that diabetes affects periodontitis in four ways. Diabetes causes a hyperinflammatory response to infection, oxidative stress, bone metabolism disorders, and increased expression of advanced glycation end products (AGEs) and their receptors (RAGE) (Lalla and Papapanou, 2011). Hyperglycemia predisposes to exaggerated inflammatory response, primes leukocytes for marginalization and superoxide production, as well as increases the free radical load in the gingival microvasculature (Gyurko et al., 2006). Cytokine dysregulation also represents the mechanism by which bacteria may induce destructive inflammatory responses in diabetic individuals (Naguib et al., 2004). Hyperglycemia can modulate the ratio of RANKL to osteoprotegerin (OPG) in periodontal tissue (Wu et al., 2014). Increased the ratio of RANKL/OPG and the secretion of AGEs, reactive oxygen species (ROS), TNF-α, IL-1β, and IL-6 in diabetes enhance periodontal ligament (PDL) and osteoblast apoptosis, induces osteoclastogenesis, and reduces bone formation (Pacios et al., 2013; Wu et al., 2015). AGEs increase IL-6 and intercellular adhesion molecule-1 (ICAM-1) expression via the RAGE, MAPK, and nuclear factor-κB (NF-κB) pathways in human gingival fibroblasts and exacerbate the progression of the pathogenesis of periodontal diseases (Nonaka et al., 2018). The AGE-RAGE interaction induces the production of ROS, the production of inflammatory cytokines such as TNF-α, and the activation of NF-κB (Brownlee, 2001). AGE-RAGE also serves as a promoting factor to regulate osteoclast formation (Ding et al., 2006).

**FIGURE 2:**
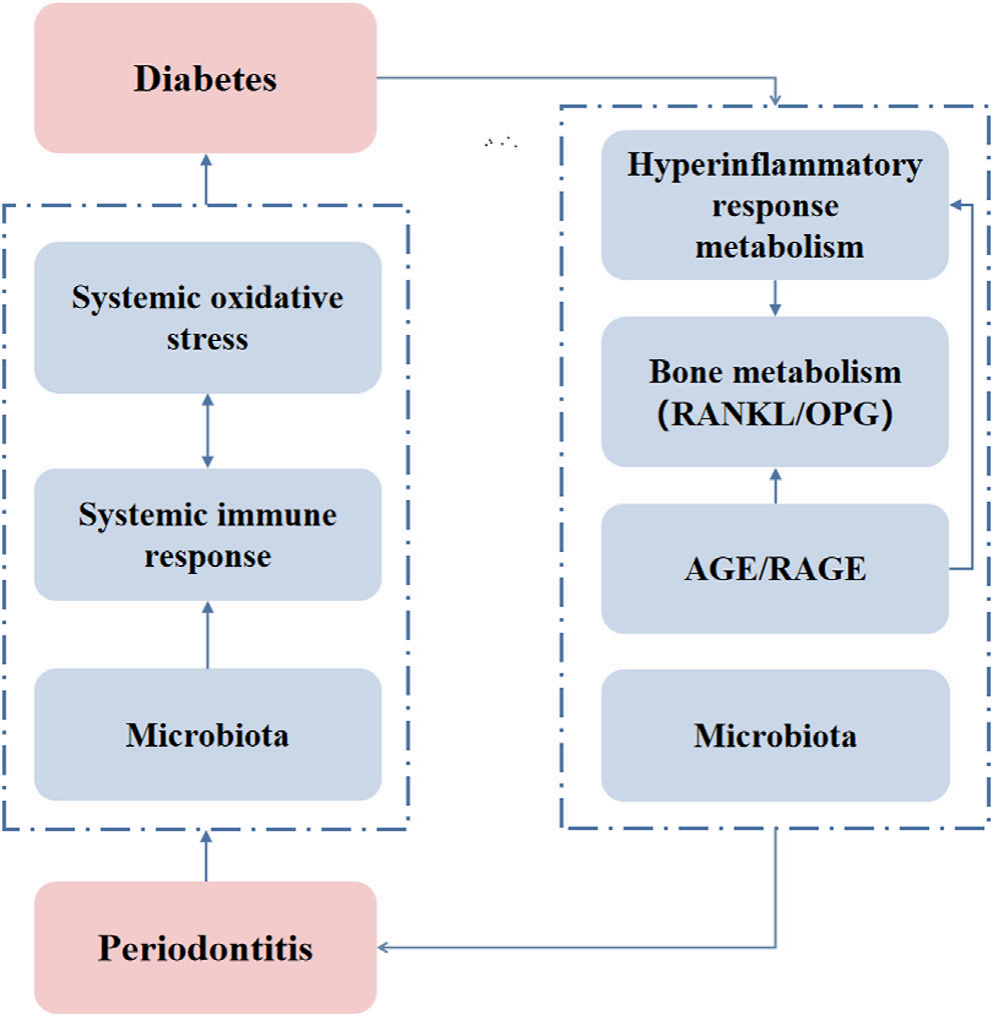
The mechanisms of the two-way relationship of periodontitis and diabetes. AGE/RAGE: advanced glycation end product/receptor of advanced glycation end products.

Oxidative stress plays a critical role in diabetes complications. Inflammation induced by increased intracellular ROS also contributes to diabetes complications (Pitocco et al., 2010). The upregulation of ROS play important role in the establishment and progression of periodontitis through the development of oxidative stress (Sczepanik et al., 2020). The increase in superoxide production activates the increase in AGE formation and RAGE expression, as well as the protein kinase C (PKC) and hexosamine pathways. Through these pathways, increased intracellular ROS cause defective angiogenesis in response to ischemia activates many proinflammatory pathways (Giacco and Brownlee, 2010). Regarding the effect of periodontitis on diabetes, it is generally believed that periodontitis induced periodontal microbiota dysbiosis. The disease concomitantly impacts the regional and systemic immune response impairing glucose metabolism (Su et al., 2013; Ilievski et al., 2015; Huang et al., 2016; Blasco-Baque et al., 2017). Inflammation markers such as c-reactive protein (CRP) and IL-6 are involved in insulin resistance, which positively correlates with the progress of diabetes (Pradhan et al., 2001). NAcht Leucine-rich repeat protein 3 (NLRP3), a pathogen recognition molecule, activates caspase-1. Activated caspase-1 cleaves and promotes the maturation of IL-1β that induces pancreatic inflammation, β-cell death, T2DM progression, and insulin-dependent diabetes development (Zhou et al., 2010). NLRP3 inflammasome-mediated effector adipose tissue T cells activation also leads to a decrease in insulin sensitivity. These cells regulate insulin resistance by releasing IFN-γ (Vandanmagsar et al., 2011). Pieces of literature had indicated that periodontitis causes an increase in systemic oxidative stress which could influence diabetes (Allen et al., 2011; Bastos et al., 2012). In addition, the microbiota plays a critical role in the connection between diabetes and periodontitis. T2DM patients are more susceptible to subgingival microbiome dysbiosis, potentially due to impaired host metabolic and immune regulation (Shi et al., 2020). Hypoglycemic therapy reconstructs the salivary microbiota of diabetes patients (Sun et al., 2020). Periodontitis affects diabetes through oral and gut microbiota dysbiosis. Gut microbial shifting driven by periodontitis contributes to systemic inflammation and metabolic dysfunction during the progression of T2DM (Li et al., 2020). Effective periodontal therapy reduced insulin resistance and improved periodontal health status and insulin sensitivity in patients with T2DM and chronic periodontitis (Mammen et al., 2017). The anti-diabetic drugs not only play an indirect role in treating inflammation due to diabetes control but also have a direct anti-inflammatory effect (Kothari et al., 2016). Figure 2 illustrates the mechanisms of two-way relationship of periodontitis and diabetes that intensify the severity of both diseases. Antibacterial drugs are often used as adjuvant treatments for patients with diabetes and periodontitis. A prospective clinical study showed the adjunct of doxycycline (Doxy) to conventional periodontal therapy provides an additional benefit in reducing the glycemic level and improves periodontal health (Das et al., 2019). Photodynamic therapy + Doxy possesses the best efficacy in lowering HbA1c% of non-smoking chronic periodontitis patients with diabetes (Cao et al., 2019). The adjunctive use of systemic metronidazole and amoxicillin improves the microbiological and clinical outcomes of scaling and root planing in subjects with chronic periodontitis and T2DM (Tamashiro et al., 2016). Adjunctive omega-3 polyunsaturated fatty acids (ω-3 PUFA) and low-dose aspirin (ASA) after periodontal debridement provides clinical and immunological benefits to periodontitis patients with T2DM (Castro Dos Santos et al., 2020). Metformin, a traditional hypoglycemic drug, has been found to have anti-inflammatory effects. Metformin reduces the activity of NLRP3 inflammasomes by inhibiting the expression of Nek7, and significantly reduces experimental diabetic periodontitis *in vivo* and *in vitro* (Zhou et al., 2019). Hypoglycemic therapy has the potential to reconstruct the salivary microbiota and improve the localized conditions of diabetes patients with periodontitis (Sun et al., 2020). There are also some uncommon drugs used to treat periodontitis-diabetes comorbidities. A double-blind clinical trial study showed that melatonin supplementation in adjunct with non-surgical periodontal therapy improves inflammatory and periodontal status in T2DM with chronic periodontitis (Bazyar et al., 2019). Locally delivered atorvastatin was found to be effective in the treatment of intrabony defects in chronic periodontitis patients with T2DM (Kumari et al., 2016). Propolis can also be used as an adjuvant drug for scaling and root planning that significantly reduces the levels of fasting plasma glucose, and serum N-(carboxymethyl) lysine, and improves the periodontal treatment effect in patients with T2DM and chronic periodontitis (El-Sharkawy et al., 2016). The above clinical studies have not clarified whether the treatment effects are through unilateral benefit to diabetes or periodontitis that indirectly affects the other side. However, it is predictable that the drugs that mitigate the inflammation or reconstruct the microbiome could be beneficial to treat periodontitis-diabetes comorbidity.

## Sulfonylureas

**FIGURE 3:**
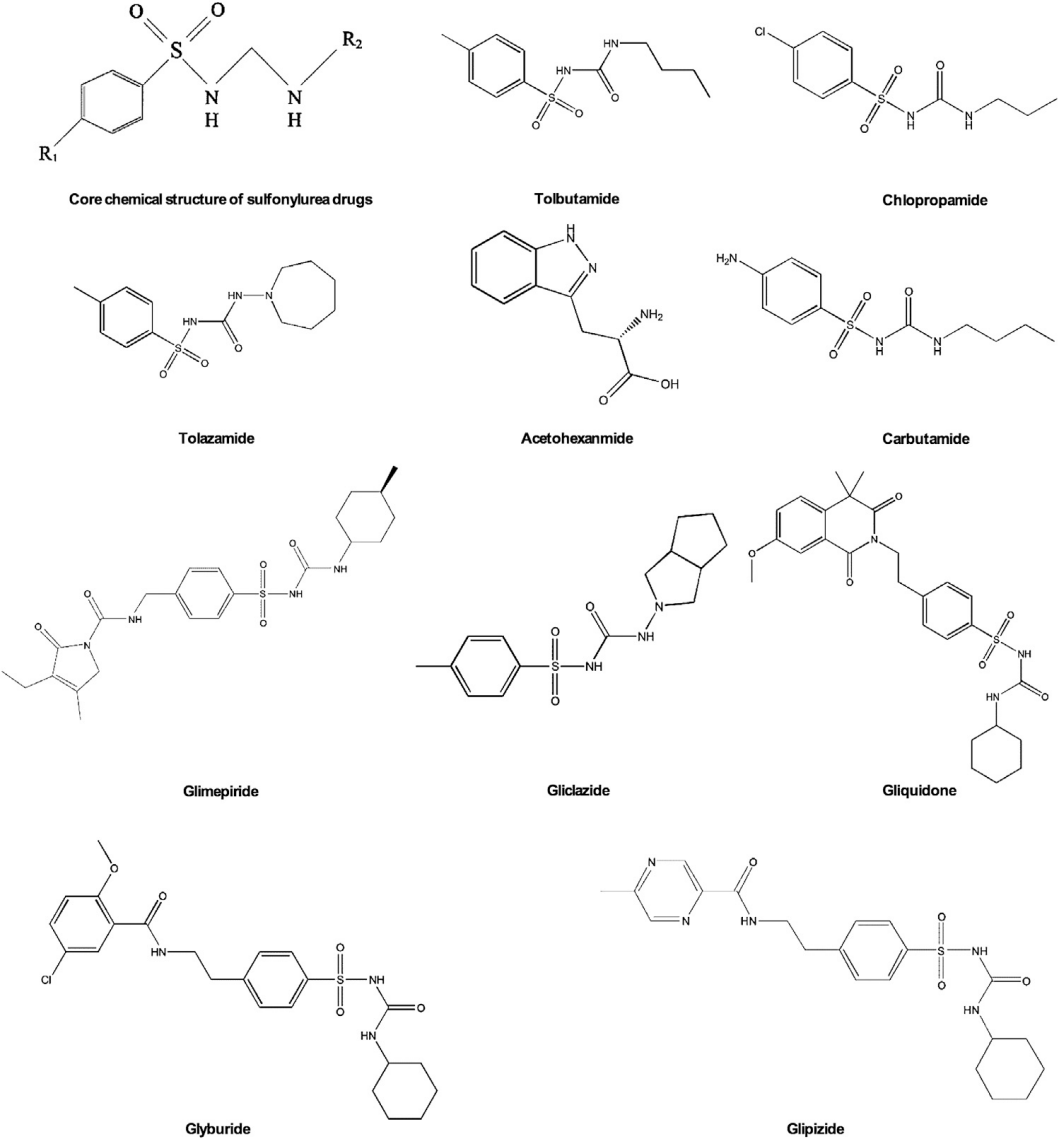
Chemical structures of sulfonylureas.

Sulfonylurea is currently one of the most common diabetes drugs. French scientist Marcel J. Janbon discovered the hypoglycemic effect of sulfa drugs in 1842. Since then, sulfonylurea has been extensively researched and developed to treat diabetes (Loubatieres-Mariani, 2007). Sulfonylurea drugs have been developed into more than ten kinds of oral hypoglycemic drugs, which are usually divided into two generations. Various sulfonylureas are derived by changing the nitrogen of the urea group and the para substituent of the benzene ring structure (Figure 3). In the first generation of sulfonylurea drugs chlorpropamide, tolbutamide, tolazamide, acetohexamide, and carbutamide the urea substituents are smaller and more polar, thus making the aryl-sulfonylurea more soluble in water. In the second generation of sulfonylurea drugs glyburide, glipizide, Gliclazide, glisoxepide, and glimepiride the substituents are large with nonpolar lipophilic groups that are easier to penetrate cell membranes and are therefore more effective (Doyle and Egan, 2003). The pancreatic β-cell membrane contains sulfonylurea receptors coupled with adenosine triphosphate (ATP)-sensitive potassium current IK(ATP) as well as voltage-dependent calcium channels. After binding with receptors sulfonylurea drugs block IK(ATP) and prevent potassium outflow, resulting in depolarization of cell membranes, enhancing voltage-dependent calcium channel opening, and extracellular calcium influx. The increased intracellular free calcium concentration triggers exocytosis and insulin release (Ashcroft and Rorsman, 2012; Lv et al., 2020). Therefore, sulfonylurea drugs only exert an effect on patients with T2DM who still have functional pancreatic β-cells in the body.

## Mechanisms of Periodontitis-Diabetes Comorbidity

Periodontitis-related systemic diseases are likely induced by three ways: hematogenous dissemination of periodontal bacteria, transfer of inflammatory mediators from periodontal tissue to blood, and oropharyngeal or oral-intestinal transfer of periodontal bacteria (Figure 1) (Teles and Wang, 2011; Mammen et al., 2020). Structural equation modeling was used to evaluate periodontal treatment effects on oral and systemic inflammation, and researchers found that diabetes and periodontitis together appear to increase systemic inflammation, with evidence of inflammation reduction following periodontal treatment (Preshaw et al., 2020). Diabetes increases inflammation in the periodontium, which causes changes in bacterial composition. Diabetes results in increased inflammation reflected by an increase in leukocytes and increased cytokine expression as well as a change in bacterial composition that enhances the overall pathogenicity of the microbiota (Graves et al., 2020).

**FIGURE 4:**
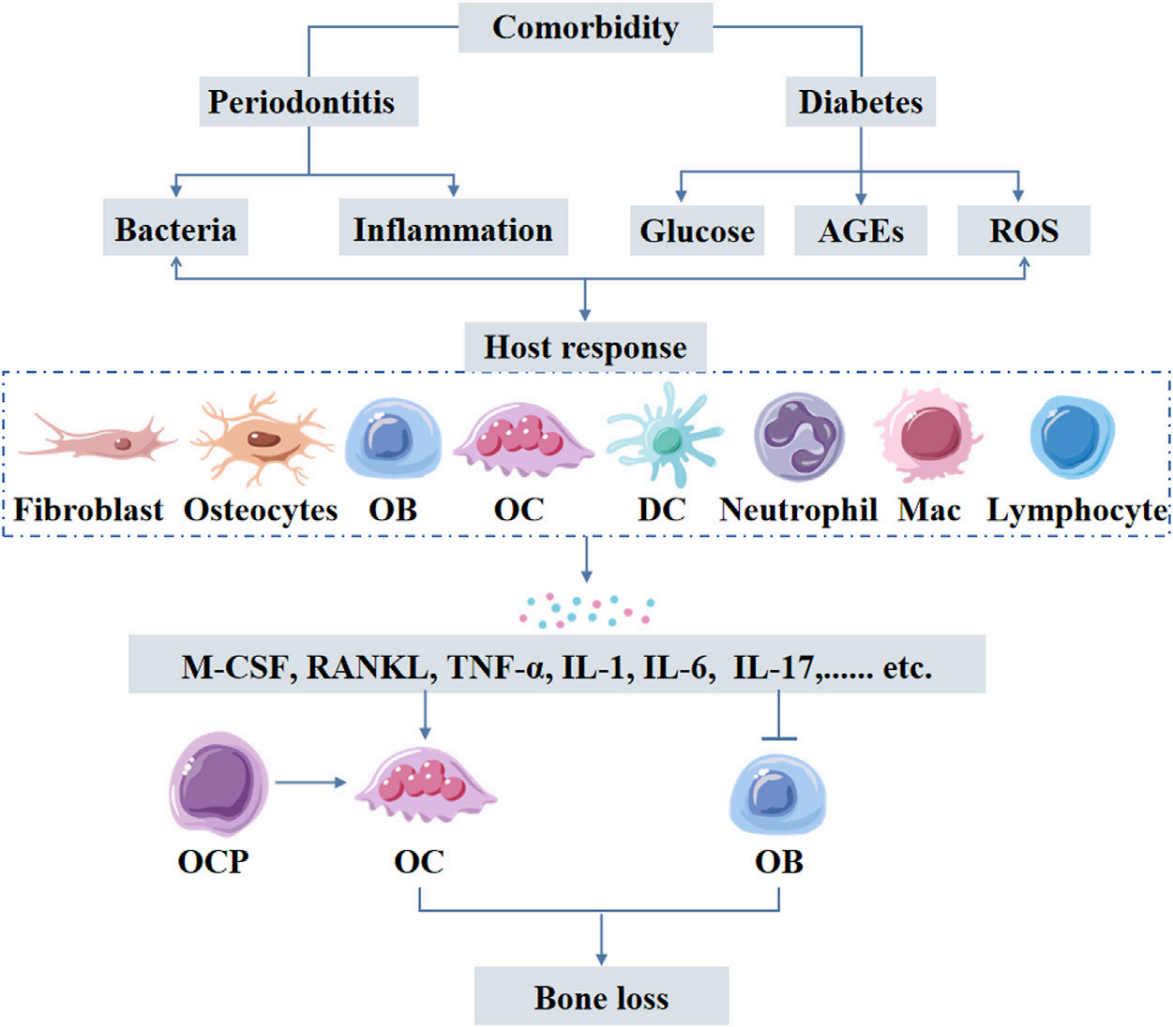
Periodontitis-diabetes comorbidity induces host response triggering the release of inflammatory mediators form variety of cells including immune cells. Elevated levels of inflammatory mediators cause both local and systemic effects. Inflammatory mediator-induced osteoclastogenesis and compromised osteoblast function are the main causes of excessive alveolar bone loss in periodontitis-diabetes comorbidity. AGEs: advanced glycation end products; ROS: reactive oxygen species; OB: osteoblasts, OCP: osteoclast precursors; OC: osteoclasts; DC: dendritic cells; Mac: Macrophages.

Hematopoietic stem cells and progenitor cells sense systemic infections or inflammation in the bone marrow through inflammatory cytokine receptors, thereby differentiate into infection-resistant cells such as neutrophils and monocytes (Teitelbaum and Ross, 2003). This process relies on IL-6 serum levels, which increase during *Porphyromonas gingivalis* (Zhao et al., 2020). Monocytic lineage is the precursor of osteoclasts and differentiates into osteoclasts by further stimulation of macrophage colony-stimulating factor (M-CSF) and RANKL (Teitelbaum and Ross, 2003). Diabetes increases the levels of glucose, AGEs, and ROS in periodontal tissues leading to increased inflammation. Intensified inflammation further impacts the oral microbiota dysbiosis and stimulates periodontal ligament fibroblasts, osteoblasts, and osteocytes to produce pro-osteoclastogenic factors. The inflammation also affects these cells to reduce bone formation, resulting in greater net bone loss (Figure 4) (Graves et al., 2020).

**FIGURE 5:**
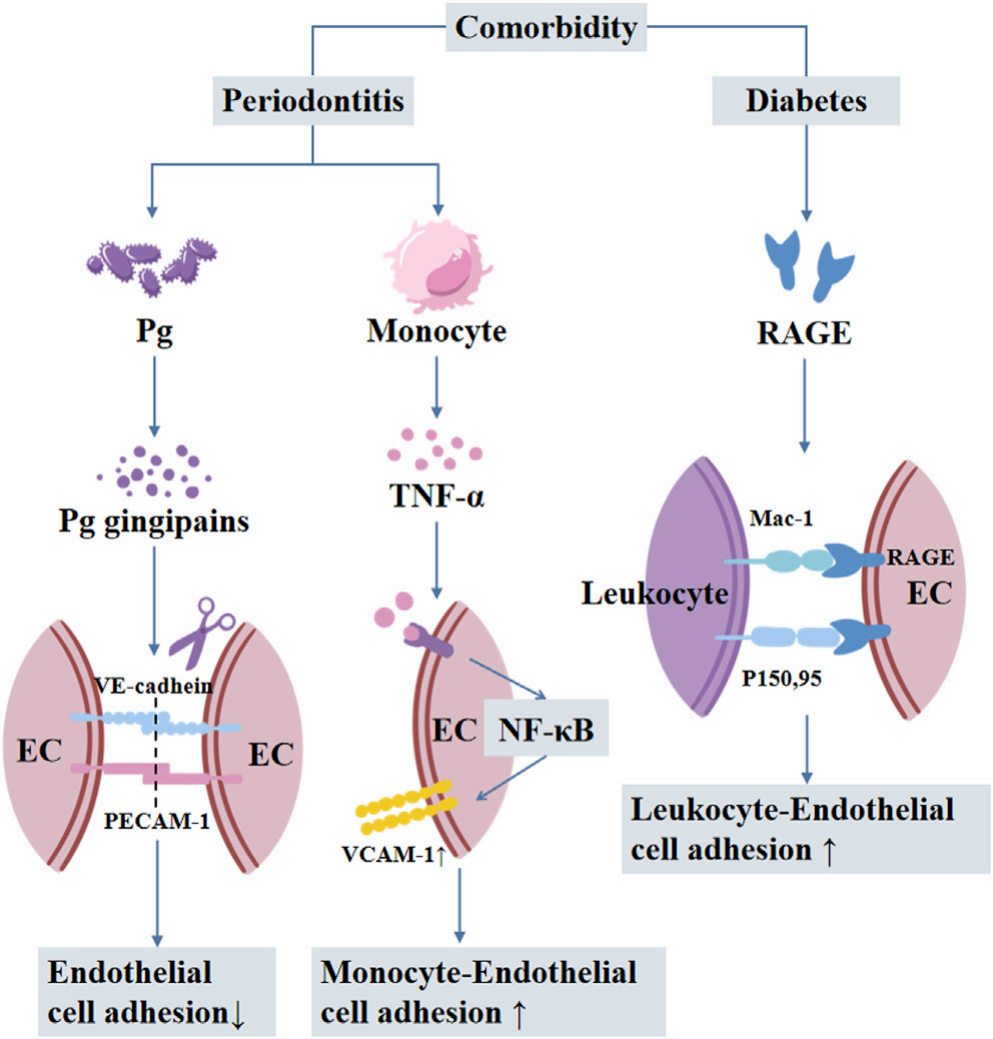
The mechanisms involved in periodontitis-diabetes comorbidity-mediated vascular inflammation and atherosclerosis. Pg: Porphyromonas gingivalis; EC: endothelial cells; RAGE: receptor of advanced glycation end products.

Vascular inflammation is attributed to periodontitis-induced activation and adhesion of circulating monocytes to aortic endothelial cells via NF-κB/p65 nuclear translocation and upregulation of VCAM1 (Miyajima et al., 2014). Local treatment of periodontitis attenuates vascular inflammation and atherosclerosis in experimental models of periodontitis (Hasturk et al., 2015). *Porphyromonas gingivalis* is the most abundant bacterial species detected in non-diseased vascular tissue of patients with atherosclerosis (Mougeot et al., 2017). Gingipains are important for the virulence of *Porphyromonas gingivalis* in extra-oral sites. *Porphyromonas gingivalis* induces edema by increasing vascular permeability, which is attributed to gingipain-dependent degradation of platelet endothelial cell adhesion molecule (PECAM1) and vascular endothelial cadherin disrupting the endothelial barrier (Farrugia et al., 2021). Periodontitis-associated insulin resistance and hyperglycemia also induce the formation of AGEs, which is involved in several metabolic disorders, including vascular inflammation (Chavakis et al., 2003), and atherosclerosis (Ruiz et al., 2020) in experimental periodontitis in diabetic mice (Figure 5) (Lalla et al., 2000).

## Anti-Inflammatory Effect of Sulfonylureas

Sulfonylureas have shown the potential to alleviate inflammation. Insulin stimulation leads to the activation of different pathways involved in metabolic regulation, including the phosphatidylinositol-3-kinase (PI3K) cascade (Kumar and Dey, 2002). Compared to the healthy gingival tissue, PI3K expression is increased in periodontal tissues affected by periodontitis (Ozcan et al., 2017) (Sarkar et al., 2012). Gliclazide reduces oxidative stress, blocks PI3K signal transmission, and reduces matrix metalloproteinase 2 (MMP-2), cathepsin K, and RANKL levels in the rat periodontitis model. Gliclazide treatment alleviates periodontitis and alveolar bone resorption (Araújo et al., 2019).

MMPs, as a class of zinc-dependent endopeptidases, are produced in large quantities when host reactions occur in a variety of pathological conditions. MMPs degrade extracellular matrix and basement membrane destroying periodontal tissue (Sorsa et al., 2006). The researchers had reported the elevated levels of MMP in the serum of patients with inflammatory periodontal tissue and gingival crevicular fluid of periodontitis patients (Silosi et al., 2015). Glyburide has been reported to reduce plasma MMP-9 levels (Sheth et al., 2016). Reducing the elevated MMP levels could mitigate the periodontitis-induced oral tissue destruction. However, the ability of various sulfonylureas to reduce the MMPs level during periodontitis still needs to be investigated.

Macrophages and monocytes are key regulators of the inflammatory process. During inflammation, IL-1β is mainly secreted by mononuclear cells and macrophages (Koh and DiPietro, 2011). Cryopyrin/NALP3/NLRP3 is an essential component of inflammasomes triggered by microbial ligands, danger-associated molecular patterns (DAMPs), and crystals. Glyburide is the first identified compound to prevent cryopyrin activation and microbial ligand-, DAMPs-, and crystal-induced IL-1β secretion (Lamkanfi et al., 2009; Masters et al., 2010). Nine different sulfonylureas have been reported to inhibit the activation of NLRP3 in mouse bone marrow-derived macrophages in a dose-dependent manner. Nano molar concentration of MCC950, glyburide, sulofenur, glimepiride, gliquidone, or glisoxepide inhibits NLRP3 activation and stimulates insulin secretion in mouse pancreatic cells, indicating the dual effects (Hill et al., 2017). Chronic inflammation often causes delayed wound healing in diabetes. Mitigation of inflammation is part of the treatment of diabetes-related complications. Glyburide promotes the inflammatory regulator-140 receptor-interacting protein ubiquitin degradation by activating Ca^2+^/calmodulin dependent protein kinase II (CamKII) that leads to anti-inflammatory M2 macrophage polarization (Lin et al., 2018). Glyburide and glimepiride have been shown to inhibit the release of IL-1β in macrophage-like THP-1 cells by targeting the activity of NLRP3 inflammasomes. Similarly, the rat model of periodontitis has shown the inhibitory effect of glyburide on inflammatory cell infiltration and alveolar bone osteoclast formation during the development of periodontitis (Kawahara et al., 2020). Glyburide inhibits NLRP3 formation, and induces the expression of pro-healing growth factors insulin-like growth factor-1 (IGF-1), TGF-β and IL-10, thereby promotes wound healing in diabetic mice (Mirza et al., 2014).

CRP is an acute-phase reactive protein and a non-specific marker of inflammation. Cross-sectional studies have shown that periodontitis and poor oral hygiene are associated with elevated serum CRP levels (Torrungruang et al., 2019). Glyburide reduces CRP level during treating diabetes (Putz et al., 2004). Clinical studies have shown that gliclazide reduces soluble ICAM-1 (sICAM-10, serum soluble E-selectin (sE-selectin), and hsCRP in diabetic patients (Räkel et al., 2007). Moreover, gliclazide blocks insulin-mediated neutrophil migration through endothelial cells and the expression of PECAM-1 by inhibiting the activation of MAPK (Okouchi et al., 2004).

There is a clear connection between diabetes and inflammation, and sulfonylurea drugs have obvious positive effects on inflammation mitigation. The literature had reported the various mechanisms of action of different sulfonylurea drugs during inflammation mitigation (Table 1).

**TABLE 1:** Effect of sulfonylureas in pathophysiology of inflammatory diseases.

Sulfonylurea	Disease	Model used	Mechanism	Reference
Gliclazide	Periodontitis	Rat periodontitis model	-Reduces oxidative stress via inhibition of PI3K signaling, MMP-2, cathepsin K, and RANKL level	Kumar and Dey, (2002); Araújo et al., (2019)
	Coronary artherosclerosis	*In vitro*	-Blocks insulin-mediated neutrophil migration through endothelial cells	Okouchi et al. (2004)
		Model of neutrophils transmigration across umbilical vein endothelial cells	-Inhibits the expression of PECAM-1 via inhibition of MAPK	
	Diabetes	Clinical trial	-Reduces sICAM-1, sE-selectin, and hsCRP	Räkel et al. (2007)
Glyburide	Brian swelling	Clinical trial	-Blocks SUR1-TRPM4 channel in neurons, astrocytes, and endothelium	Sheth et al. (2016)
			-Reduces MMP-9 plasma level	
	The inflammation in diabetes	*In vitro*	-Inhibits NLRP3 inflammasomes	Mirza et al. (2014); Hill et al. (2017); Kawahara et al. (2020)
		THP-1	-Activates Ca^2+^/CamKII	
			-Induce the expression of pro-healing growth factors: IGF-1, TGF-β, and IL-10	
	Diabetes	Clinical trial	- Reduces CRP level	Putz et al. (2004)
MCC950	__	*In vitro*	-Inhibits NLRP3 at nanomolar concentrations	Hill et al. (2017)
		BMDM		
Sulofenur	__	*In vitro*	-Inhibits NLRP3 at nanomolar concentrations	Hill et al. (2017)
		BMDM		
Glimepiride	__	*In vitro*	-Inhibits NLRP3 at nanomolar concentrations	Hill et al. (2017)
		BMDM		
Gliquidone	__	*In vitro*	-Inhibits NLRP3 at nanomolar concentrations	Hill et al. (2017)
		BMDM		
Glisoxepide	__	*In vitro*	-Inhibits NLRP3 at nanomolar concentrations	Hill et al. (2017)
		BMDM		

BMDM: bone marrow derived macrophages.

## The Effect of Sulfonylurea Drugs on Bone Metabolism

Periodontitis eventually destroys periodontal tissues and alveolar bone leading to tooth looseness and even loss. The main reason for alveolar bone loss in periodontitis is the imbalance of bone metabolism, mainly due to the increased osteoclasts’ number and activity (Straka et al., 2015). Activation of nuclear factor-kappa B receptor activator (RANK) signaling in osteoclasts precursors during bacterial infection is a key driver of enhanced osteoclast number and activity in periodontitis. Bacterial components induce the production of proinflammatory cytokines TNF-α and IL-1β, which enhance the activation of RANK by RANKL and down-regulating OPG signaling (Darveau, 2010).

In the rat periodontitis model, oral administration of glyburide significantly inhibits the infiltration of inflammatory cells and osteoclasts in the periodontal tissue (Kawahara et al., 2020). MCC950, a diarylsulfonylurea compound, which is the most specific inhibitor of NLRP3 inflammasome (Laliberte et al., 2003), inhibits the classical and non-classical pathways of NLRP3 inflammasome activation (Coll et al., 2015). The administration of MCC950 prevents alveolar bone loss in aging mice, but not in young mice. *In vitro* studies have shown that MCC950 treatment directly inhibits osteoclast differentiation (Zang et al., 2020). These effects are achieved through the inhibition of NLRP3 inflammasome and IL-1β expression by sulfonylurea drugs. Table 2 summarizes the effect of sulfonylureas on bone metabolism.

**TABLE 2:** Sulfonylurea-mediated effects on bone metabolism and vascular functions.

Sulfonylurea	Effect	Model used	Mechanism	Reference
MCC950	Suppress alveolar bone loss in aged mice	Aged mice and *In vitro* BMDM culture	Reduces caspase-1 activation	Zang et al. (2020)
Glimepiride	Protect estrogen deficiency- mediated bone loss	Ovariectomized rats	Reverses the inhibitory effect of high glucose on the PI3K/Akt/eNOS pathway	Ma et al. (2011); Ma et al. (2014)
	Inhibits the formation of atheromatous plaques in high-cholesterol fed rabbits	*In vitro* HCAECs culture	Induces the production of NO in HCAEC by activating PI3K/Akt resulting vasodilation	Ueba et al. (2005)
	Improves the proliferation and migration of VSMC	*In vitro* HASMC culture	KATP channels	Zhang et al. (2019)
Glyburide	Significantly suppresses the infiltration of the number of osteoclasts in the alveolar bone	Periodontitis rat model	Suppresses activation of the NLRP3 inflammasome	Kawahara et al. (2020)
	Changes the circulating bone biomarkers	Clinical trial	Reduces osteoclast markers	Zinman et al. (2010)
	Enhances long-term brain repair and improves behavioral recovery	MCAO rat model	Acute blockade of SUR1 and enhanced angiogenesis	Ortega et al. (2013)
	Improves the proliferation and migration of VSMC	*In vitro*	KATP channels	Zhang et al. (2019)
		HASMC culture		
Gliclazide	Inhibits the retinal endothelial cell proliferation and VEGF expression	*In vitro*	Inhibits AGE-induced activation of PKC-, MAPK- and NF-κB signaling	Mamputu and Renier, (2004)
		BREC culture		
	Protects vascular endothelial cells from apoptosis	*In vitro* HUVECs culture	Attenuate oxidative stress induced by high glucose in HUVECs	Corgnali et al. (2008)
	Attenuates the sustained inflammatory process that occurs in the atherosclerotic plaque	*In vitro*	-Reduces oxLDL-, and AGEs-induced monocyte adhesion to ECs	Renier et al. (2003)
		Model of neutrophils transmigration across HUVECs	-Inhibits EC adhesion molecule expression and NF-κB activation	
	Reduces oxidative stress in patients with T2DM	Clinical trial	Improves plasma antioxidant status related to NO-mediated vasodilation enhancement	Fava et al. (2002)
	Prevents vascular obstructive diseases in T2DM	*In vitro*	Shows an inhibitory effect on VSMC	Zhang et al. (2019)
		HASMCs culture		
Glipizide	Inhibits the growth and metastasis of cancer	TRAMP transgenic mouse model	Reduces microvessel density in PC tumor tissues	Qi et al. (2016)
		*In vitro* HUVECs culture	-inhibits the tubular structure formation of HUVECs by regulating the HMGIY/Angiopoietin-1 signaling pathway	

BMDM: bone marrow derived macrophages; NO: nitric oxide.

Sulfonylurea drugs stimulate pancreatic β-cells to increase insulin secretion. Insulin is a bone anabolic factor that acts through insulin receptor substrate (IRS) signaling and glucose uptake. *In vivo* studies suggest that the absence of IRS gene decreases bone mass, reduces osteoblast/osteoclast function, and impairs bone turnover (Ogata et al., 2000). IRS-2 signal maintains the tendency of bone formation to exceed bone resorption, and IRS-1 signal maintains bone renewal. The integration of these two signals leads to strong bone anabolic effects of insulin and IGF-1 (Akune et al., 2002). The reduction of bone mass in most diabetic patients is related to the function of β-cells. Moreover, *in vitro* studies have shown that insulin regulates bone metabolism by stimulating osteoblast proliferation and inhibiting osteoclast activity (Kalaitzoglou et al., 2019). Therefore, the sulfonylureas that can stimulate insulin secretion may also play a role in making bone metabolism tend to the direction of bone formation.

Many studies have proven that bone metabolism and energy metabolism are mutually regulated by closely related mechanisms. OPG induces islet β-cell replication by regulating CREB and GSK3 pathways (Kondegowda et al., 2015). Under the same pathological conditions, hyperglycemia has various adverse effects on glucose metabolism. Diabetes affects bone through glucose metabolism disorders, destruction of bone microvascular function, and muscle endocrine function, which is also a unique bone feature in diabetes (Lecka-Czernik, 2017). Hyperglycemia interferes with the proliferation and mineralization of osteoblasts. Studies have shown that hyperglycemia inhibits the PI3K/Akt/eNOS pathway in rat osteoblasts, thereby interfering with the osteoblast transcription factors such as RUNX2, which have an important effect on osteoblast differentiation. Glimepiride stimulates the proliferation and differentiation of rat osteoblasts through the PI3K/Akt/eNOS signaling pathway (Ma et al., 2014). Glimepiride reverses the inhibitory effect of high glucose on the PI3K/Akt/eNOS pathway, enhances osteoblast proliferation, expression of RUNX2, osteocalcin, and alkaline phosphatase mRNA, and protects rat mandibular osteoblast-apoptosis from high concentrations of glucose (Ma et al., 2011).

Studies have shown that glimepiride alleviates the skeletal damage caused by estrogen deficiency in ovariectomized rats and promotes bone formation (Fronczek-Sokol and Pytlik, 2014). There is also a gender-wise difference in the effect of glyburide on bone metabolism. The results of clinical sampling and the diabetes endpoint progression test showed that after glyburide treatment, bone resorption marker type I collagen c-terminal peptide (CTX) is significantly reduced in women but not in men. Glyburide alters the bone formation markers: type 1 procollagen amino-terminal peptide (P1NP), and bone alkaline phosphatase (bsALP). The effect of glyburide had been reported as changes of +0.2% P1NP in males and −11.6% bsALP in females (Zinman et al., 2010).

There is still considerable disagreement about the association of sulfonylurea drugs with fracture risk. Some studies have shown that compared with thiazolidinediones, sulfonylurea-treated patients have a lower fracture risk (Vestergaard et al., 2005; Dormuth et al., 2009; Solomon et al., 2009), and the same results have been obtained in cross-sectional surveys in postmenopausal populations (Kanazawa et al., 2010). However, some studies have found that there is no significant correlation between sulfonylurea treatment and fracture risk (Melton et al., 2008; Colhoun et al., 2012). These controversial results may be due to differences in study design, patient age/sex, drug concentration, and treatment duration. Multicenter clinical studies using the same protocol are needed to confirm the inconsistent outcome of these studies.

## The Effect of Sulfonylurea Drugs on Angiogenesis

Angiogenesis is an important process that occurs under physiological and pathological conditions. Inflammatory diseases are one of the angiogenesis-dependent diseases. Inflammation-induced angiogenesis affects many diseases, and periodontitis is one of them (Carmeliet, 2003). The formation of blood vessels is strictly regulated and this process involves the synergistic effect between various cells, cytokines, growth factors, and extracellular matrix components (Andreuzzi et al., 2017). Among them, vascular endothelial growth factor (VEGF) is the essential factor for vascular endothelium, which is also an important factor in the pathogenesis of invasive periodontitis and chronic periodontitis (Unlü et al., 2003). Gingival tissue, gingival crevicular fluid, and serum show a high expression trend of VEGF in periodontitis (Noguchi et al., 2011; Pradeep et al., 2011). VEGF also interacts with factors that regulate bone homeostasis and bone development, affecting bone metabolism (Peng et al., 2002). Under high glucose conditions, VEGF inhibits insulin secretion of pancreatic β-cells, sulfonylurea receptor expression, and inwardly rectifying potassium channel gene 6.2 (Kir6.2) expression (An et al., 2017). Gliclazide reduces protein kinase C (PKC) translocation, extracellular signal-regulated protein kinase 1/2, and NF-κB induced by AGEs in the bovine retinal endothelial cell (BREC), inhibiting BREC proliferation and VEGF expression (Mamputu and Renier, 2004).

H_2_S and C-type Natriuretic Peptide (CNP) also act as vasoactive agents and induce angiogenic responses. H_2_S enhances EC migration through a KATPchannel/p38/hsp27 pathway. Blockade of KATP channels in ECs by glibenclamide blocked H2S-induced EC migration (Papapetropoulos et al., 2009). Similarly, CNP mediates angiogenesis via KATP activation. Subsequent research pointed out that ATP-sensitive potassium channel activation induces angiogenesis effects *in vitro* and *in vivo* (Khambata et al., 2011). KATP activation and the expression of the Kir6.1 KATP subunit may underpin angiogenesis to a variety of vasoactive stimuli, including H2S, VEGF, and CNP (Umaru et al., 2015). Sulfonylurea drugs may have inhibitory effects on angiogenesis via targeting Kir6.1 KATP subunit.

The effects of sulfonylurea drugs on angiogenesis have been proven by multiple animal experiments. Table 2 summarizes the effect of sulfonylureas on vascular functions. Male Wistar rats treated with low-dose glyburide simulate stroke after middle cerebral artery occlusion treatment. Histological evaluation showed that glyburide treatment enhances angiogenesis in the hippocampus (Ortega et al., 2013). Under the stimulation of angiogenesis, MMP-9 degrades multimerin 2 (MMRN2). The decreased MMRN2 acts as a negative feedback regulator to promote the expression of VEGFA and angiogenesis (Andreuzzi et al., 2017). In periodontitis, MMP-9 is up-regulated in serum and gingival crevicular fluid (Yang et al., 2019). Intravenous glyburide shows a decrease in plasma MMP-9 during a clinical study on the safety and efficacy of glyburide in the treatment of brain swelling after cerebral infarction (Sheth et al., 2016). These reports suggest that glyburide may have an inhibitory effect on angiogenesis by reducing the MMP-9 and modulating MMRN2/VEGFA pathway.

Anti-diabetes drugs also have a direct effect on tumor growth. The second-generation sulfonylurea glipizide has obvious vascular inhibitory effects and inhibits tumor-induced angiogenesis by up-regulating natriuretic peptide that inhibits tumor growth and metastasis (Qi et al., 2014; Qi et al., 2016). The results of the studies on the effects of sulfonylurea drugs on angiogenesis are not consistent. Some studies have shown that glyburide inhibits vascular function. However, the anabolic effect of sulfonylurea drugs on vascular function cannot be denied (Matsuzawa et al., 2015). Different sulfonylurea drugs have different effects on vascular function. The different study models and drugs used might be responsible for the contradictory results.

Gliclazide attenuates oxidative stress induced by high glucose in human umbilical vein endothelial cells and protect vascular endothelial cells from apoptosis (Corgnali et al., 2008). Gliclazide also reduces the adhesion of monocytes and endothelial cells induced by oxidized low-density lipoprotein (oxLDL) and AGEs *in vitro*. This effect reduces the ability of monocytes adhesion in the blood vessel walls and the continuous inflammatory process that occurs in atherosclerotic plaques (Renier et al., 2003). Similar events might occur during periodontitis. However, future studies are needed to support this hypothesis.

Glimepiride induces the production of nitric oxide (NO) in human coronary artery endothelial cells by activating PI3K/Akt, and more NO release will play a role in vasodilation (Ueba et al., 2005). Gliclazide reduces oxidative stress in patients with T2DM by improving plasma antioxidant status, and this effect is also related to NO-mediated vasodilation enhancement (Fava et al., 2002). Sulfonylurea receptors are also present in vascular smooth muscle cells (VSMC), and KATP channels play an important role in the proliferation and migration of VSMC induced by glyburide and glimepiride. In contrast, gliclazide has an inhibitory effect on VSMC, which makes gliclazide a potential therapeutic to prevent vascular obstructive diseases in T2DM (Zhang et al., 2019).

## Summary

**FIGURE 6:**
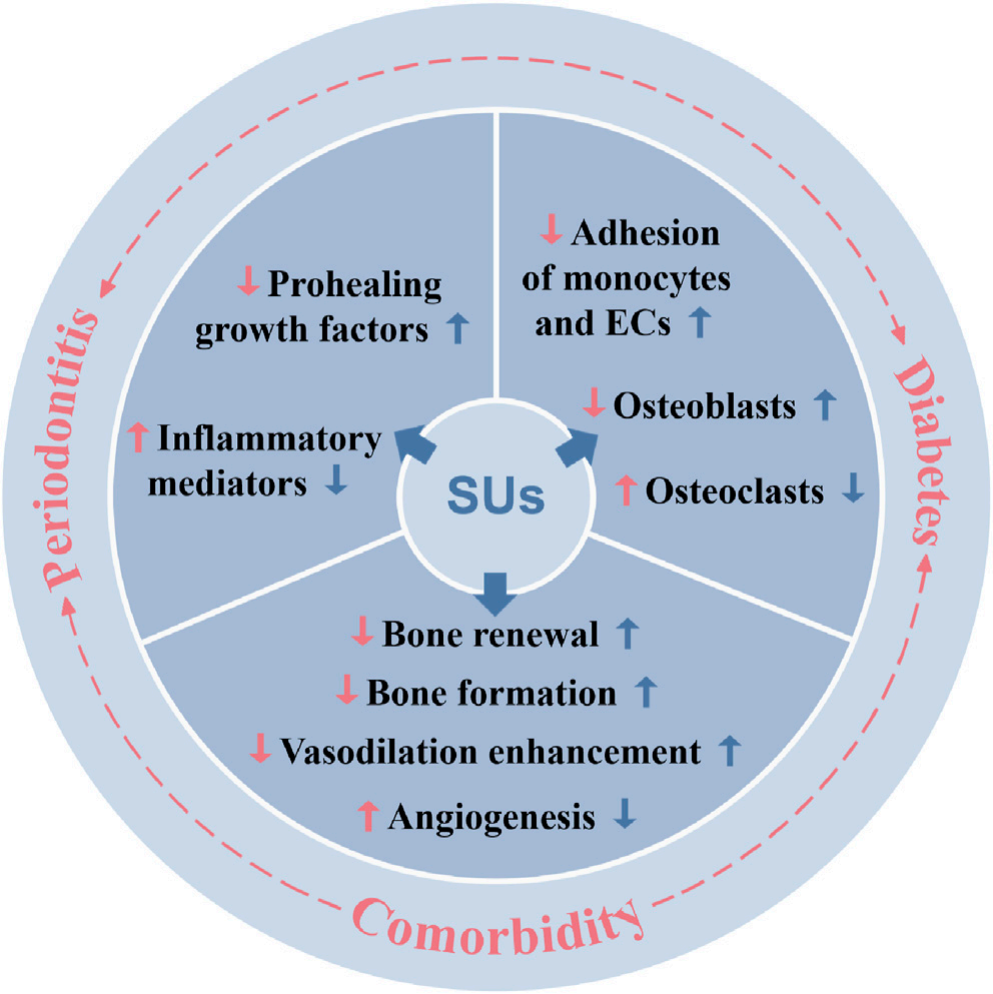
Periodontitis, diabetes, and periodontitis-diabetes comorbidity affects various local and systemic functions, and sulfonylureas had shown potential to alleviate those effects. SUs: sulfonylureas; ECs: endothelial cells.

In summary, sulfonylurea drugs have multiple therapeutic potential other than the traditional treatment of diabetes. The occurrence and development of periodontal disease are closely related to inflammatory factors, bone metabolism balance, and neovascularization. Affected by systemic metabolic changes, periodontitis combined with diabetes aggravates the pathology of both diseases. Sulfonylurea drugs alleviate periodontitis possibly via direct mitigation of inflammation or indirectly via anti-diabetic effect (Figure 6). The above-mentioned studies indicated that sulfonylurea drugs are likely to have a therapeutic effect by directly acting on periodontitis suggesting its possible application to treat periodontitis alone. Moreover, sulfonylureas can be used as a “one stone two birds” to treat periodontitis-diabetes comorbidity. However, more *in vitro* and *in vivo* studies are needed to test the efficacy of sulfonylureas on periodontitis or periodontitis-diabetes comorbidity and to unravel the underlying mechanisms of action.

## Abbreviations

PI3K, phosphatidylinositol-4,5-bisphosphate3-kinase; MMP, matrix metalloproteinase; PECAM-1, platelet endothelial cell adhesion molecule-1; MAPK, mitogen-activated protein (MAP) kinase; sICAM-1, soluble intercellular adhesion molecule-1; hsCRP, high-sensitivity C-reactive protein; SUR1-TRPM, sulfonylurea receptor 1-transient receptor potential melastatin 4; NLRP3, NLR family pyrin domain containing 3; THP-1, human monocytic leukemia cell line; BMDM, bone marrow-derived macrophages; Ca2+/CamKII, Ca^2+^/calcium calmodulin-dependent kinase II; IGF-1, insulin-like growth factor 1; TGF-β, transforming growth factor-β; IL-10, interleukin-10; HCAECs, human coronary artery endothelial cells; HASMCs, human aortic smooth muscle cells; VSMC, vascular smooth muscle cell; VEGF, vascular endothelial growth factor; MCAO, middle cerebral artery occlusion; KATP, ATP sensitive potassium (K) channel; oxLDL, oxidized low-density lipoprotein; AGE, advanced glycation end-products; PKC, protein kinase C; MAPK, mitogen-activated protein kinase; NF-κB, nuclear factor kappa B; HUVECs, human umbilical vein endothelial cells; ECs, endothelial cells
